# The Degree of Hydroxylation of Phenolic Rings Determines the Ability of Flavonoids and Stilbenes to Inhibit Calcium-Mediated Membrane Fusion

**DOI:** 10.3390/nu15051121

**Published:** 2023-02-23

**Authors:** Polina D. Zlodeeva, Egor V. Shekunov, Olga S. Ostroumova, Svetlana S. Efimova

**Affiliations:** Laboratory of Membrane and Ion Channel Modeling, Institute of Cytology of Russian Academy of Sciences, Tikhoretsky 4, 194064 Saint Petersburg, Russia

**Keywords:** viral fusion inhibitors, membrane, lipid vesicles, polyphenol nutrients, piceatannol, taxifolin, catechin, lipid-packing stress

## Abstract

This paper discusses the possibility of using plant polyphenols as viral fusion inhibitors with a lipid-mediated mechanism of action. The studied agents are promising candidates for the role of antiviral compounds due to their high lipophilicity, low toxicity, bioavailability, and relative cheapness. Fluorimetry of calcein release at the calcium-mediated fusion of liposomes, composed of a ternary mixture of dioleoyl phosphatidylcholine, dioleoyl phosphatidylglycerol, and cholesterol, in the presence of 4′-hydroxychalcone, cardamonin, isoliquiritigenin, phloretin, resveratrol, piceatannol, daidzein, biochanin A, genistein, genistin, liquiritigenin, naringenin, catechin, taxifolin, and honokiol, was performed. It was found that piceatannol significantly inhibited the calcium-induced fusion of negatively charged vesicles, while taxifolin and catechin showed medium and low antifusogenic activity, respectively. As a rule, polyphenols containing at least two OH-groups in both phenolic rings were able to inhibit the calcium-mediated fusion of liposomes. In addition, there was a correlation between the ability of the tested compounds to inhibit vesicle fusions and to perturb lipid packing. We suggest that the antifusogenic action of polyphenols was determined by the depth of immersion and the orientation of the molecules in the membrane.

## 1. Introduction

Polyphenol phytochemicals are classified into different classes such as monophenols, flavonoids, phenolic acids, and other nonflavonoid polyphenolics [1]. Flavonoids are the most explored and extensive subclass of natural polyphenolic compounds [2]. The main prospect for their clinical use lies in their bioavailability and ubiquity in nature as a result of the accumulation of flavonoids in plants as a response to stress for acclimation by external adverse conditions [3,4]. Based on the structural features of the fragment linking two phenolic rings, flavonoids are divided into several subclasses: chalcones, flavones, isoflavones, flavonols, flavanones, flavononols, flavan-3-ols, and anthocyanins [5].

**Figure 1:**
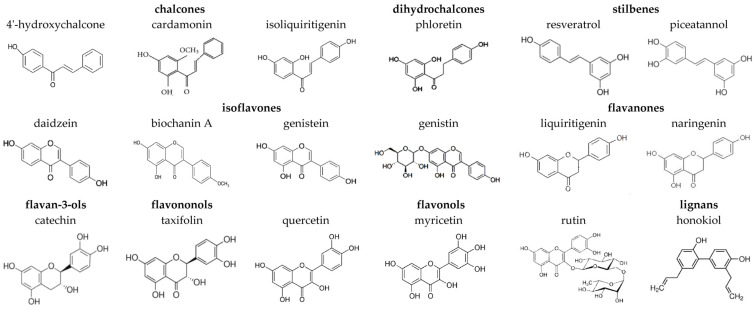
Chemical structures of tested plant polyphenols.

Chalcones and dihydrochalcones are known as open-chain unsaturated and saturated flavonoids, respectively [6]. These compounds are mainly found in hops, citrus fruits, apples, tomatoes, potatoes, licorice, and cardamom [7,8]. 4′-hydroxychalcone (Figure 1) shows potent activity against human coronaviruses [9]. Chalcone cardamonin (Figure 1) demonstrates multiple promising healthcare properties against cancer, cardiovascular diseases, diabetes, neurological disorders, inflammation, rheumatoid arthritis, etc. [10]. Isoliquiritigenin is another important anticancer chalcone [11,12]. Dihydrochalcone phloretin (Figure 1) is thought to have a variety of therapeutic benefits against cancers, diabetes, liver injury, kidney injury, encephalomyelitis, ulcerative colitis, asthma, arthritis, and cognitive impairment [13].

Nonflavonoid stilbenes have a structure similar to that of chalcones but have two phenolic tails linked by ethanol or ethylene. Resveratrol (Figure 1), commonly found in grapes, peanuts, and berry fruits, shows significant activity against cancer, cardiovascular diseases, and neurodegenerative disorders [14]. The 3′-hydroxylated derivative of resveratrol, piceatannol (Figure 1), found in various plants, shows enhanced anticancer and antibiofilm activity [14].

Isoflavones have a B ring connected to a C ring at the C3 position. They are almost exclusively found in leguminous plants [15]. The key soy isoflavonoids are genistein, daidzein, biochanin A (Figure 1), and their glycosides [16,17]. The beneficial effects of isoflavones and soy-rich diets include reducing the risk of cardiovascular disease and bone disorder [18,19].

The structural features of flavones are differentiated by a double bond between C2 and C3 and the carbonyl group at the C4 position. Flavonols are a class of C3-hydroxylated flavones. The main food sources of flavonols are onions, red wine, olive oil, berries, and grapefruits [20]. Quercetin and its glycoside rutin (Figure 1) are the most common flavonoids in the diet. Quercetin has pronounced antibacterial properties including activity against MRSA and *Staphylococcus epidermidis* [21]. Another flavonol, myricetin (Figure 1), has been shown to have a therapeutic effect on many diseases, including tumors of different types, inflammatory diseases, atherosclerosis, thrombosis, cerebral ischemia, diabetes, Alzheimer’s disease, and pathogenic microbial infections [22].

Flavanones retain a structure identical to that of flavones, with the only difference being the absence of a double bond in the C ring. Key sources of flavanones are citrus fruits, lemons, oranges, and grapes [6]. The flavanones liquiritigenin and naringenin (Figure 1) are thought to be neuroprotective [23,24].

Flavanonols have a basic structure similar to that of flavanones and a hydroxyl group at C3 position. A widespread flavanonol taxifolin (Figure 1) is of interest due to its wide range of health benefits including promising anti-inflammatory, antimicrobial, and anticancer activity; it is also active against cardiovascular and liver diseases [25].

The flavonoids abundant in tea, namely, flavan-3-ols or catechins, are characterized by a saturated bond between C2 and C3, C3-hydroxylation, and unoxidized C4. The chemical structure of catechin is presented in Figure 1. Catechins have attracted attention due to their antihypertensive, antibacterial, anti-inflammatory, and antitumor activity [26].

Lignans are plant nonflavonoid polyphenolic compounds composed of two or more phenylpropanoid units. Lignan honokiol (Figure 1) is one of the natural extracts of *Magnolia officinalis,* which was proved to have diverse pharmacological activities, including anti-inflammation, antitumor, and neuroprotective effects [27], and is potent against skin diseases [28].

In addition to the health benefits described above, many plant polyphenols have antiviral activity affecting all stages of the virus life cycle: entry, reproduction, and budding [29]. For example, piceatannol and cardamonin inhibit the penetration and reproduction of human immunodeficiency virus (HIV) in host cells, which is probably due to their binding to the viral surface glycoprotein gp120 [30,31]. Several studies have attributed the antiviral activities of phloretin and biochanin A to their ability to modulate the PI3K/AKT/NF-kB (phosphatidylinositol 3-kinase/protein kinase B/nuclear factor kappa-light-chain-enhancer of activated B cells) signaling pathway, which, in turn, regulates the balance between apoptosis and the virus-induced synthesis of proinflammatory cytokines [32,33,34,35,36,37]. Licochalcone A exhibits inhibitory activity at an early stage of enterovirus A71 replication [38]. The data [39] indicate that baicalein prevents human cytomegalovirus entry by affecting the kinase activity of the epidermal growth factor receptor. It was reported that quercetin blocks virus penetration by interacting with membrane glycoproteins such as the glycoprotein D (gD) of herpes simplex virus and neuraminidase (NA) of influenza A virus [40]. The results reported by Peng et al. [41] showed that myricetin exhibits antiviral activity against avian infectious bronchitis virus by inhibiting the deubiquitinating activity of papain-like protease. Kaempferol glycosides can block the 3a channel of coronaviruses, preventing the production of the virus [42]. Emodin exhibits antiviral activity against coronavirus by disrupting S-protein interaction with the angiotensin-converting enzyme 2 receptor (ACE2) [43]. Quercetin, epigallocatechin gallate, and gallocatechin gallate show good inhibition of the 3C-like protease of coronavirus [44].

Constant variability of the viral genome sets limits on the pool of viral targets, increasing the risk of cross-resistance during treatment with targeted antiviral drugs [45]. Thus, the relevance of searching for agents that affect specific constant viral structures is undeniable [46], and the facultative lipid envelope of the virion is an obvious candidate as a target for potentially novel antiviral drugs, because it is a conserved part of the virus. Plant flavonoids are excellent candidates for such antiviral drugs due to their amphiphilic nature and ability to interact with the lipid microenvironment [47,48,49].

The fusion process is sensitive to changes in the properties of fusing membranes [50,51]. It is known that secondary plant metabolites can inhibit virus fusion with the cell by varying the physical characteristics of the membrane. In particular, cepharanthine has a membrane-stabilizing effect and inhibits the fusion of HIV with the cell by reducing bilayer fluidity [52]. The ability of alkaloids to inhibit viral fusion is related to their lipid-associated disordering action [53].

Flavonoids might also modulate the fusion process by altering the lipid bilayers of membranes. For example, naringenin, rutin, genistein, genistin, and biochanin A and some of their metabolites (equol, 4-hydroxyequol, dihydrodaidzein, and dihydrogenistein) are able to decrease membrane fluidity [49]. Resveratrol has a dual membrane effect similar to that of cholesterol [54]. Authors showed that this flavonoid can either increase membrane thickness or decrease it, depending on the initial lipid order and organizational state. Biochanin A and phloretin were shown to disrupt lipid-ordered domains due to their fluidizing effect, while myricetin did not demonstrate such ability [55]. Butein, 4′-hydroxychalcone, cardamonin, liquiritigenin, naringenin, and resveratrol demonstrated the ability to disorder dipalmytoylphosphocholine and promote membrane positive curvature stress [56].

The aim of this study was to assess the ability of plant polyphenols, in particular, chalcones (4′-hydroxychalcone, cardamonin, and isoliquiritigenin), dihydrochalcones (phloretin), stilbenes (resveratrol and piceatannol), isoflavones (daidzein, biochanin A, genistein, and genistin), flavanones (liquiritigenin and naringenin), flavan-3-ols (catechin), flavononols (taxifolin), and lignans (honokiol), to inhibit the calcium-mediated fusion of negatively charged lipid vesicles and to compare their antifusogenic activity with that of flavonols (quercetin, myricetin, and rutin) that have been previously studied. The role of the OH hydroxylation of the aglycone structure in the ability to disorder membrane lipids and to suppress liposome fusion was evaluated.

## 2. Materials and Methods

### 2.1. Materials

CaCl_2_, NaCl, NaOH, calcein, Triton X-100, Sephadex G-50, 4-(2-Hydroxyethyl)piperazine-1-ethanesulfonic acid (HEPES), dimethylsulfoxide (DMSO), and plant polyphenols: chalcones (4′-hydroxychalcone (≥99%, high-performance liquid chromatography (HPLC)), cardamonin (≥98%, HPLC), isoliquiritigenin (≥98%, HPLC)), dihydrochalcones (phloretin (≥99%, HPLC)), stilbenes (resveratrol (≥99%, HPLC), piceatannol (≥98%, HPLC)), isoflavones (daidzein (≥98%, HPLC), biochanin A (≥95%, HPLC), genistein (≥98%, HPLC), genistin (≥95%, HPLC)), flavanones (liquiritigenin (≥97%, HPLC), naringenin (≥95%, HPLC)), flavan-3-ols (catechin (≥98%, HPLC)), flavononols (taxifolin (≥90%, HPLC)), flavonols (quercetin, myricetin, and rutin), and lignans (honokiol (≥98%, HPLC)) were purchased from Sigma-Aldrich Company Ltd. (Gillingham, United Kingdom).

Lipids synthetic 1,2-dioleoyl-*sn*-glycero-3-phospho-(1-*rac*-glycerol) (DOPG), 1,2-dioleoyl-*sn*-glycero-3-phosphocholine (DOPC), 1,2-dipalmitoyl-*sn*-glycero-3-phospho-(1-*rac*-glycerol) (DPPG), 1,2-dipalmitoyl-*sn*-glycero-3-phosphocholine (DPPC), and cholesterol (CHOL) were obtained from Avanti Polar Lipids (Avanti Polar Lipids, Inc., Alabaster, AL, USA).

### 2.2. Calcein Leakage Assay

The calcein fusion assay was performed on a Fluorat-02-Panorama spectrofluorometer (Saint-Petersburg, Russia) with excitation wavelength 490 nm and emission 520 nm. Small unilamellar vesicles (Ø100 nm) composed of DOPG/DOPC/CHOL (40/40/20 mol.%) were formed by extrusion using an Avanti Polar Lipids^®^ mini-extruder (Avanti Polar Lipids, Inc., Alabaster, AL, USA). The initial suspension of lipids in trichloromethane with a total lipid concentration of 3 mM was dried under a weak stream of nitrogen until a lipid film was formed. The obtained film was hydrated by a buffer (35 mM calcein, 10 mM HEPES, pH 7.4), and a cycle of 5 repetitions of freezing–thawing was performed. This liposome suspension was then passed 13 times through a polycarbonate membrane with a pore diameter of 100 nm using a mini-extruder to obtain unilamellar vesicles of the target size. The calcein not captured by liposomes was removed from the solution by gel filtration in a column filled with Sephadex G-50. The calcein within the vesicles experiences self-quenching at millimolar concentrations, whereas the luminescence of the calcein efflux depends on the level of mixing of the internal content of fusing liposomes [57].

The fusion of DOPG/DOPC/CHOL (40/40/20 mol.%) liposomes was induced with 40 mM CaCl_2_ [58,59]. Liposomes were incubated with 20 µM polyphenols for 30 ± 10 min before the addition of calcium. The tested polyphenols at the used concentrations did not produce noticeable (more than ~13%) calcein leakage in the absence of a fusion inducer.

Total calcein leakage due to the destruction of all liposomes was measured at the end of each experiment after addition of detergent, Triton X-100, at a concentration of 1%.

The relative fluorescence of calcein efflux from liposomes showing the percentage of fusion (*RF*) was calculated using the following equation:

(1) $$RF=\frac{I-I_{0}}{\frac{I_{max}}{0.9}-I_{0}}\cdot 100\%$$

where *I* and *I*_0_ are fluorescence intensities in the presence of both calcium and polyphenol and in the presence of polyphenol alone, respectively; *I_max_* is the intensity after the addition of Triton X-100. To calculate the dilution of the sample by detergent, a coefficient of 0.9 was introduced.

The antifusogenic activity of polyphenols was described by relative inhibition, *RI*:

(2) $$RI=\frac{RF\left(Ca\right)-RF(Ca\_P)}{RF(Ca)}\cdot 100\%$$

where *RF*(Ca) and *RF*(Ca_P) are the maximum relative fusion produced by CaCl_2_ in the absence and presence of tested polyphenols, respectively.

The kinetics of calcein release at the liposome fusion was described by a one-exponential function with time constant, τ.

All experiments were performed at room temperature (25 ± 1 °C).

### 2.3. Differential Scanning Microcalorimetry

Differential scanning microcalorimetry experiments were performed with a μDSC 7EVO microcalorimeter (Setaram, Caluire-et-Cuire, France). Giant unilamellar vesicles composed of 1,2-dipalmitoyl-*sn*-glycero-3-phosphocholine (DPPC) or 1,2-dipalmitoyl-*sn*-glycero-3-phospho-(1-*rac*-glycerol) (DPPG) were prepared by the electroformation method using Vesicle Pre Pro^@^ (Nanion Technologies, Munich, Germany) (standard protocol, 3 V, 10 Hz, 58 min, 55 °C). The resulting DPPC or DPPG liposome suspension contained 2.5 mM lipid and was buffered by 5 mM HEPES at pH 7.4. The molar lipid:polyphenol ratio was equal to 10:1. The liposomal suspension was heated and cooled at a constant rate of 0.2 and 0.3 °C/min, respectively. The reversibility of the thermal transitions was assessed by reheating the sample immediately after the cooling step from the previous scan. The temperature dependence of the excess heat capacity was analyzed using Calisto Processing (Setaram, Caluire-et-Cuire, France).

The peaks on the thermograms were characterized by the pretransition peak (*T_p_*), the maximum temperature of the main phase transition (*T_m_*), and the width of the main peak, i.e., the temperature difference between the upper (onset) and lower (completion) boundary of the main phase transition (Δ*T_b_*) of DPPC or DPPG.

### 2.4. Statistical Processing of Results

The values of *RF*, τ, *RI*, Δ*T_p_*, Δ*T_m_*, and ΔΔ*T_b_* were averaged from 3 to 7 independent experiments and are presented as mean ± standard error (*p* ≤ 0.05).

To prove the statistical significance of the detected differences in the *RF*, *T_m_*, and Δ*T_b_* values before and after the addition of tested polyphenols, the nonparametric Mann–Whitney–Wilcoxon test was used (*—*p* ≤ 0.01).

## 3. Results and Discussion

**Figure 2:**
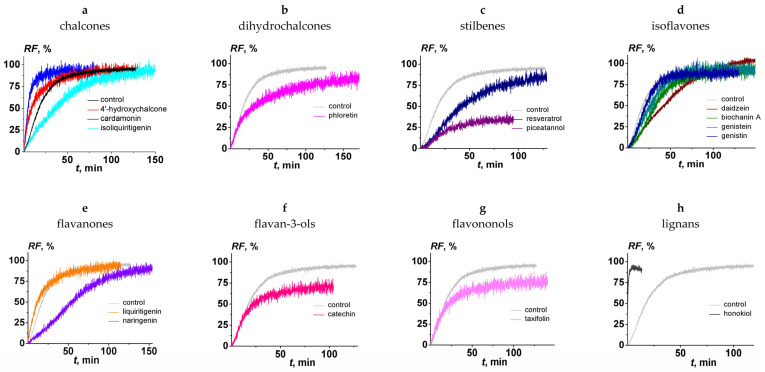
The time dependence of relative fluorescence of calcein (*RF*) leaked by the fusion of DOPC/DOPG/CHOL (40/40/20 mol.%) vesicles induced by 40 mM CaCl_2_ in the absence (black line in figure (**a**) and grey in figure (**b**–**h**) and presence of polyphenols: (**a**) 4′-hydroxychalcone, cardamonin, and isoliquiritigenin; (**b**) phloretin; (**c**) resveratrol and piceatannol; (**d**) daidzein, biochanin A, genistein, and genistin; (**e**) liquiritigenin and naringenin; (**f**) catechin; (**g**) taxifolin; and (**h**) honokiol. Liposomes were incubated with 20 μM polyphenols for 30 min before the addition of inductor. The relationship between the colored line and the polyphenols is given in the figure.

The effect of the chalcones (4′-hydroxychalcone, cardamonin, and isoliquiritigenin); dihydrochalcones (phloretin); stilbenes (resveratrol and piceatannol); isoflavones (daidzein, biochanin A, genistein, and genistin); flavanones (liquiritigenin and naringenin); flavan-3-ols (catechin); flavononols (taxifolin); and lignans (honokiol) on the fusion of negatively charged liposomes, composed of DOPC/DOPG/CHOL (40/40/20 mol.%) and mediated by CaCl_2_ (40 mM), was tested. Figure 2 presents the kinetics of calcein fluorescence due to vesicle fusion in the absence (black line) and presence (colored lines) of different polyphenols. The beginning of the fusion process is characterized by a sharp increase in the kinetics of calcein release, after which the graph plateaus. The maximum value of the relative fluorescence of calcein efflux from liposomes (*RF*) depended on the type of tested polyphenol (Table 1). The time dependencies of *RF* were fit by exponential functions. The values of the parameter characterizing calcein release kinetics at the liposome fusion, τ, in the absence and presence of tested polyphenols, varied between 10 and 70 min, except for honokiol (value decreased by an order of magnitude). The pronounced change in the fusion kinetics in the presence of honokiol can be explained by the presence of two side hydrocarbon chains, in contrast with the other tested compounds. (Figure 1).

Table 1 summarizes the maximum values of *RF* in the absence and presence of the polyphenols tested here and flavonols (quercetin, myricetin, and rutin) that were previously investigated [60]. In the absence of polyphenols, the *RF* value was equal to 92%. The percentage of fusion increased in the following order: quercetin (about 10%) < piceatannol ≈ myricetin (about 30%) < taxifolin (about 60%) ≤ catechin (about 70%) ≤ honokiol ≈ liquitigenin ≈ phloretin ≈ naringenin ≈ genistin ≈ isoliquiritigenin ≈ rutin ≈ genistein ≈ 4′-hydroxychalcone ≈ biochanin A ≈ resveratrol ≈ cardamonin ≈ daidzein (75–95%). To compare the antifusogenic activity of different polyphenols, the *RI* was calculated using the obtained *RF* values (Table 1). We concluded that 4′-hydroxychalcone, cardamonin, isoliquiritigenin, phloretin, resveratrol, daidzein, biochanin, genistein, genistin, liquiritigenin, naringenin, rutin, and honokiol did not practically inhibit the fusion of DOPC/DOPG/CHOL liposomes (*RI* did not exceed 20%). Catechin and taxifolin demonstrated moderate antifusogenic activity: *RI* was equal to about 20 and 40%, respectively (Table 1). Piceatannol significantly inhibited calcium ion-induced liposome fusion, and its *RI* was about 70%. Moreover, the previously obtained data indicated that the myricetin and quercetin were characterized by more pronounced ability to inhibit calcium-mediated fusion of DOPG/DOPC/CHOL liposomes at the same concentrations: *RI* was about 60% and 85%, respectively (Table 1 [60]). Some plant alkaloids are also characterized by a pronounced ability to inhibit the calcium-mediated fusion of negatively charged liposomes [53].

Analyzing the data presented in Table 1, we postulate that:

(1) Low hydroxylated aglycones, having 1–3 OH-groups in the structure (Figure 1), do not inhibit the CaCl_2_-mediated fusion of DOPC/DOPG/CHOL liposomes. This concerns all tested chalcones (4′-hydroxychalcone, cardamonin, and isoliquiritigenin), dihydrochalcone phloretin, isoflavones (daidzein, genistein, and biochanin A), flavanones (liquiritigenin and naringenin), and lignan honokiol.
(2) Flavonoid glycosides (genistin and rutin) are not able to inhibit the fusion of negatively charged lipid vesicles.
(3) Highly hydroxylated aglycones, containing 5–6 OH-groups (Figure 1), in particular, flavan-3-ol catechin, flavononol taxifolin, and flavonols, quercetin, and myricetin, are able to inhibit liposome fusion.
(4) Not only the number but also the localization of hydroxyl groups is important: dihydrochalcone phloretin and stilbene piceatannol, both characterized by the presence of 4 OH groups in the structure (Figure 1), exhibit different antifusogenic activities. This indicates that the degree of hydroxylation of the phenolic rings flanking the molecule plays a fundamental role. Thus, polyphenols containing at least two OH groups in both phenolic rings are able to inhibit calcium-mediated liposome fusion.
(5) The presence of a carbonyl group and a double bond in the heterocycle of active polyphenols (Figure 1) does not matter, which indicates that the conformation of the polyphenol molecule, and, in particular, its rigidity and planarity, are not the determining factors for the manifestation of antifusogenic activity by polyphenols. For example, both the flavan-3-ol catechin (planar, [61]) and the flavonol taxifolin (nonplanar [62]) are capable of inhibiting the fusion of negatively charged membranes.

**Figure 3:**
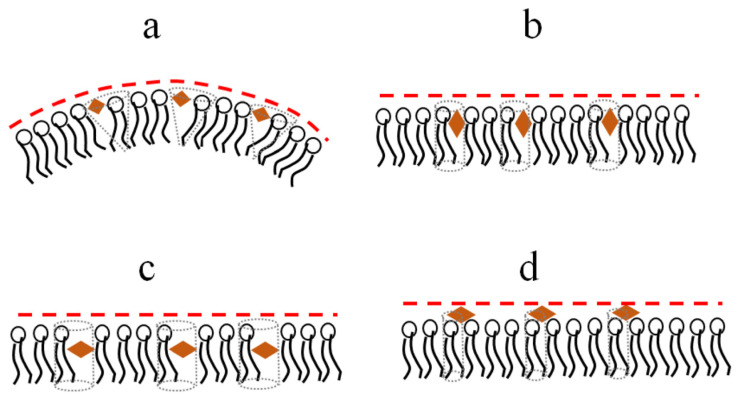
Schematic representation of the immersion of different polyphenols (orange diamonds) into membrane and their influence on lipid packing and curvature stress (**a**) Piceatannol, (**b**) Genistein, (**c**) Isoliquiritigenin, (**d**) Genistin.

The presence of at least two hydroxyl groups in both phenolic rings of the molecules of polyphenols should prevent them from being immersed in the hydrocarbon region of the membrane. The insertion of polyphenols between lipid heads can induce a disordering and positive curvature stress (Figure 3a). This should lead to the suppression of membrane fusion due to an increase in the energetic cost of formation of fusion intermediates of highly negative spontaneous curvature [50]. The polyphenols, characterized by an imbalance between the hydrophilicity of opposite parts of the molecule, orient themselves perpendicular to the normal to the bilayer, which is accompanied by a less pronounced effect on the lipid packing and curvature stress (Figure 3b). More hydrophobic molecules might incorporate deeper into the membrane and disorder membrane lipids (Figure 3c). Relatively hydrophilic glycosides (Table 1) are probably unable to significantly permeate the bilayer and modulate the transmembrane distribution of lateral pressure (Figure 3d). The assumption about the different localizations of various polyphenols in the bilayer is consistent with the data on reducing the intensity of the fluorescence of the lipid probe with a fluorophore attached to the head, 1,2-dipalmitoyl-*sn*-glycero-3-phosphoethanolamine-N-lissamine rhodamine B sulfonyl, by highly hydroxylated myricetin, while this is not the case for more hydrophobic phloretin and biochanin A [55].

To test the assumption, we performed differential scanning calorimetry of the melting of the two major components of the used lipid mixture, DPPC and DPPG, in the presence of the polyphenols possessing antifusogenic activity: piceatannol, catechin, and taxifolin. Inactive genistin was also tested for comparison. The effects of flavonols, quercetin, myricetin, and rutin had been previously analyzed [60,63].

**Figure 4:**
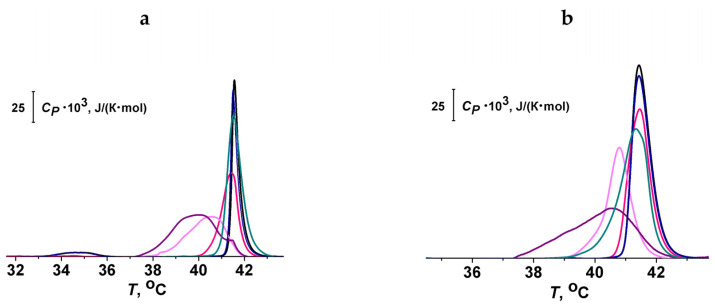
Heating thermograms of DPPC (**a**) and DPPG (**b**) (the dependence of specific heat at constant pressure, Cp, on the temperature, T) in the absence (black curves) and presence of polyphenols: piceatannol (purple curves), genistein (dark cyan curves), genistin (dark blue curves), catechin (pink curves), and taxifolin (magenta curves). The lipid:polyphenol molar ratio was equal to 10:1.

Figure 4a,b demonstrate the effects of piceatannol, genistein, genistin, catechin, and taxifolin at the lipid:polyphenol molar ratio of 10:1 on the heating thermograms of DPPC and DPPG liposomes, respectively. The thermograms were characterized by *T_p_* (temperature of pretransition), *T_m_* (the temperature of the main phase transition), and Δ*T_b_* (the width of the main peak). It can be seen that piceatannol and taxifolin had the strongest effect on the thermotropic characteristics of lipids, decreasing *T_m_* and increasing *T_b_*, while genistin had practically no effect on either DPPC or DPPG phase transition. The values of changes in the thermotropic characteristics of DPPC and DPPG melting in the presence of isoliquiritigenin, phloretin, piceatannol, catechin, taxifolin, genistein, genistin, liquiritigenin, quercetin, myricetin, and rutin are summarized in Table 2.

Glycosides had a much weaker effect on the thermotropic behavior of both lipids compared with aglycones. This finding is consistent with the lower LogD values of glycosides (Table 1) and the assumption that the aglycones are more deeply immersed into the membrane than glycosides. Aglycones characterized by similar LogD values had various effects on the *T_m_* and Δ*T_b_* values. This indicates the significance of the orientation of the polyphenolic molecule in the membrane. Probably, the predominant orientation of the long axis of the polyphenol molecule perpendicular to the normal of the bilayer should lead to a more pronounced increase in the area per lipid molecule.

The effects of piceatannol, genistein, genistin, liquiritigenin, catechin, taxifolin, and rutin on the melting of DPPC were similar or stronger than on the phase transition of DPPG. Quercetin and myricetin were characterized by more pronounced effects on DPPG melting than on DPPC phase transition. This finding might be related to the highly negative charge of the molecules of the flavonols (Table 2) and an electrostatic repulsion with negatively charged DPPG.

As expected, polyphenols’ ability to suppress membrane fusion (Table 1) is not directly related to their disordering effect (Table 2). This is in good agreement with the idea that membrane disordering is related to a general increase in the area per lipid molecule, while membrane fusion is exclusively inhibited by inducing positive curvature stress.

## 4. Conclusions

(i) Polyphenols containing at least two OH groups in both phenolic rings, in particular, piceatannol, catechin, and taxifolin, are able to inhibit the calcium-mediated fusion of DOPC/DOPG/CHOL liposomes.
(ii) The inhibitory activity of polyphenols is inter-related with their ability to influence lipid packing and curvature stress.
(iii) The ability of polyphenols to suppress membrane fusion depends on the depth of immersion and the orientation of molecules in the bilayer. Polyphenols, which are predominantly localized in the region of the lipid heads, are characterized by significant antifusogenic activity. The insertion of polyphenols induces a disordering and a positive curvature stress, which leads to the suppression of membrane fusion due to an increase in the energetic cost of the formation of fusion lipid intermediates of high negative spontaneous curvature.

## Figures and Tables

**Table 1:** The parameters characterizing the effect of polyphenols on the calcein leakage at the fusion of the DOPC/DOPG/CHOL (40/40/20 mol.%) vesicles mediated by calcium.

Polyphenol Group	Compound	LogD ^#^	RF, %	RI, %
			92 ± 3	
chalcones	4′-hydroxychalcone	3.46	92 ± 5	−4 ± 7
	cardamonin	3.64	95 ± 3	−8 ± 5
	isoliquiritigenin	3.47	84 ± 13	5 ± 15
dihydrochalcones	phloretin	3.79	81 ± 6	8 ± 7
stilbenes	resveratrol	3.37	94 ± 3	−7 ± 5
	piceatannol	3.06	29 ± 10 *	67 ± 12
isoflavones	daidzein	2.43	95 ± 4	−8 ± 6
	biochanin A	2.95	94 ± 1	−7 ± 4
	genistein	2.79	89 ± 7	−1 ± 9
	genistin	0.63	83 ± 10	6 ± 12
flavanones	liquiritigenin	2.34	79 ± 17	10 ± 20
	naringenin	2.70	82 ± 10	7 ± 11
flavan-3-ols	catechin	1.79	68 ± 8 *	22 ± 10
flavononols	taxifolin	1.65	58 ± 16 *	37 ± 17
flavonols	quercetin ^$^	1.46	9 ± 1 *	85 ± 2
	myricetin ^$^	1.06	31 ± 3 *	58 ± 4
	rutin ^$^	−1.35	88 ± 1	−5 ± 2
lignans	honokiol	5.19	75 ± 17	17 ± 20

^#^ the values of the logarithm of octanol/water distribution coefficient at pH 7.4 (LogD) were predicted with ChemAxon. ^$^—according to [60]. *—*p* ≤ 0.01 (Mann−Whitney−Wilcoxon’s test, inductor alone vs. inductor + polyphenol).

**Table 2:** The parameters characterizing the effect of the polyphenols on thermogram phase behavior.

		DPPC			DPPG	
Polyphenol	Charge ^#^	Δ*T_p_,* °C	−Δ*T_m_,* °C	ΔΔ*T_b_,* °C	−Δ*T_m_,* °C	ΔΔ*T_b_,* °C
isoliquiritigenin	−0.38	–^@^	1.7 ± 0.2	4.7 ± 0.4	ND	ND
phloretin	−0.21	–^@^	1.2 ± 0.3	5.1 ± 0.7 *	ND	ND
piceatannol	−0.14	–^@^	1.8 ± 0.2 *	4.5 ± 0.1 *	0.8 ± 0.2 *	4.8 ± 1.0 *
genistein	−0.32	0	0.2 ± 0.1	0.7 ± 0.1	0.1 ± 0.1	0.8 ± 0.2
genistin	−0.11	0	0	0.1 ± 0.1	0	0.2 ± 0.1
liquiritigenin	−0.30	–^@^	0.9 ± 0.6 ^§^	2.0 ± 0.2	0.4 ± 0.1	1.5 ± 0.2
catechin	−0.03	–^@^	0.3 ± 0.1 *	1.3 ± 0.2 *	0	0.6 ± 0.1
taxifolin	−0.35	–^@^	0.9 ± 0.1 *	1.4 ± 0.2 *	0.7 ± 0.2 *	1.3 ± 0.3 *
quercetin	−0.80	–^@,&^	0.3 ± 0.1 *^,&^	3.2 ± 0.3 *	1.2 ± 0.2 *^,$^	1.0 ± 0.1 *
myricetin	−0.88	–^@,&^	0.3 ± 0.1 *^,&^	2.8 ± 0.4 *	1.1 ± 0.2 *^,$^	0.8 ± 0.1 *
rutin	−0.48	0 ^&^	0 *^,&^	0.6 ± 0.1	0 ^$^	0.3 ± 0.1

^#^ the values of the electrical charge were predicted with ChemAxon. Δ*T_p_*, Δ*T_m_*—the changes in the positions of pretransition and main transition peaks of DPPC or DPPG in the presence of polyphenols at the lipid:polyphenol molar ratio of 10:1. *T_p_* of pure DPPC was equal to 34.8 °C; *T_m_* of pure DPPC (DPPG) was equal to 41.5 (41.4) °C. ΔΔ*T_b_*—the change in the width of the main peak in the presence of polyphenols. In the absence of polyphenols, Δ*T_b_* of pure DPPC (DPPG) was equal to 0.4 ± 0.1 (0.7 ± 0.1) °C. ND—not determined. ^@^—the pretransition was suppressed. ^&^—according to [63]. ^$^—according to [60]. ^§^—according to [56] at the lipid:polyphenol molar ratio of 9:1. *—*p* ≤ 0.01 (Mann–Whitney–Wilcoxon’s test, control vs. polyphenol).

## Data Availability

Not applicable.
